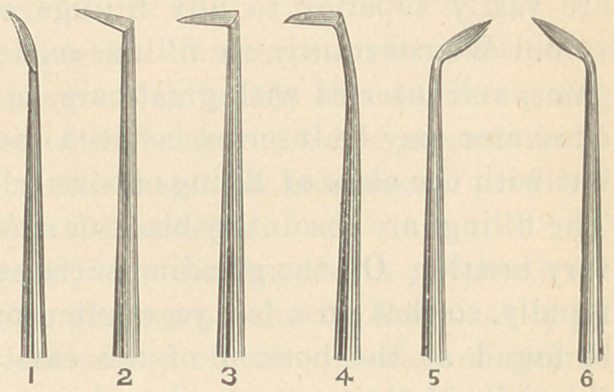# Scalers and Root Trimmers

**Published:** 1885-10

**Authors:** E. S. Talbot

**Affiliations:** Chicago, Ill.


### SCALERS AND ROOT TRIMMERS.

BY E. S. TALBOT, M. D., D. D. S., CHICAGO, ILL.

"A workman is known by the tools he uses," is a phrase applicable as well to the dentist as to the mechanic.

It refers not only to the construction of the instruments with reference to the work to be accomplished, but also to the condition they are in when needed. The specialty of dentistry being for the most part manipulative, requires numerous and varied instruments. Compare the dental instruments of a quarter of a century ago with those of to-day. They were characterized by large handles and ill-shaped shanks and blades. The delicate operations performed now would have been an impossibility in the days of primitive dental instruments. The more delicate the instrument, when properly shaped and tempered so as to have a sharp edge and not break, the easier and more successful will the results be. Perhaps in no other particular is this more noticeable than in the instruments used in removing tartar from the teeth. They should be well tempered, and so shaped that the operator, standing outside the oral cavity, may carry the instrument into the mouth, and reach all parts without materially changing his position. They should also be so constructed as to accomplish the most work possible without changing for others. A set of scalers which I have used for years with great satisfaction, and which may facilitate the operations of other practitioners, I present to the readers of the INDEPENDENT PRACTITIONER. The blades are triangular, and the shanks so shaped that the greatest surface of tartar may be reached. The blade passes readily under the gum, and between the teeth the approximal surfaces of two teeth may be reached without the removal of the instrument. Then by turning the instrument to the right or left, the cutting edge will come in contact with the free surfaces and the tartar will be removed. These instruments are constructed so as to be used toward or from the gum, having three cutting edges. For the preparation of roots of teeth for the reception of gold crowns and bands, I use Nos. 1, 2, and 3 exclusively, No. 3 particularly being employed. The instruments are tempered quite hard, and with the Arkansas stone a very sharp cutting edge may be obtained. By carrying them under the gum the enamel and dentine may be shaved off quickly and without injury to the soft tissues.

## Figures and Tables

**Figure f1:**